# Supplementary material for: Deciphering Genetic Alterations of Hairy Cell Leukemia and Hairy Cell Leukemia-like Disorders in 98 Patients
Source: Cancers (Basel). 2022 Apr 10;14(8):1904. doi: 10.3390/cancers14081904 (PMC9028144; doi:10.3390/cancers14081904)
Supplement: Supplementary file 1 [file cancers-14-01904-s001.zip › cancers-1638957-Supplementary/cancers-1638957-supplementary-figures.pdf]

cancers

MDPI

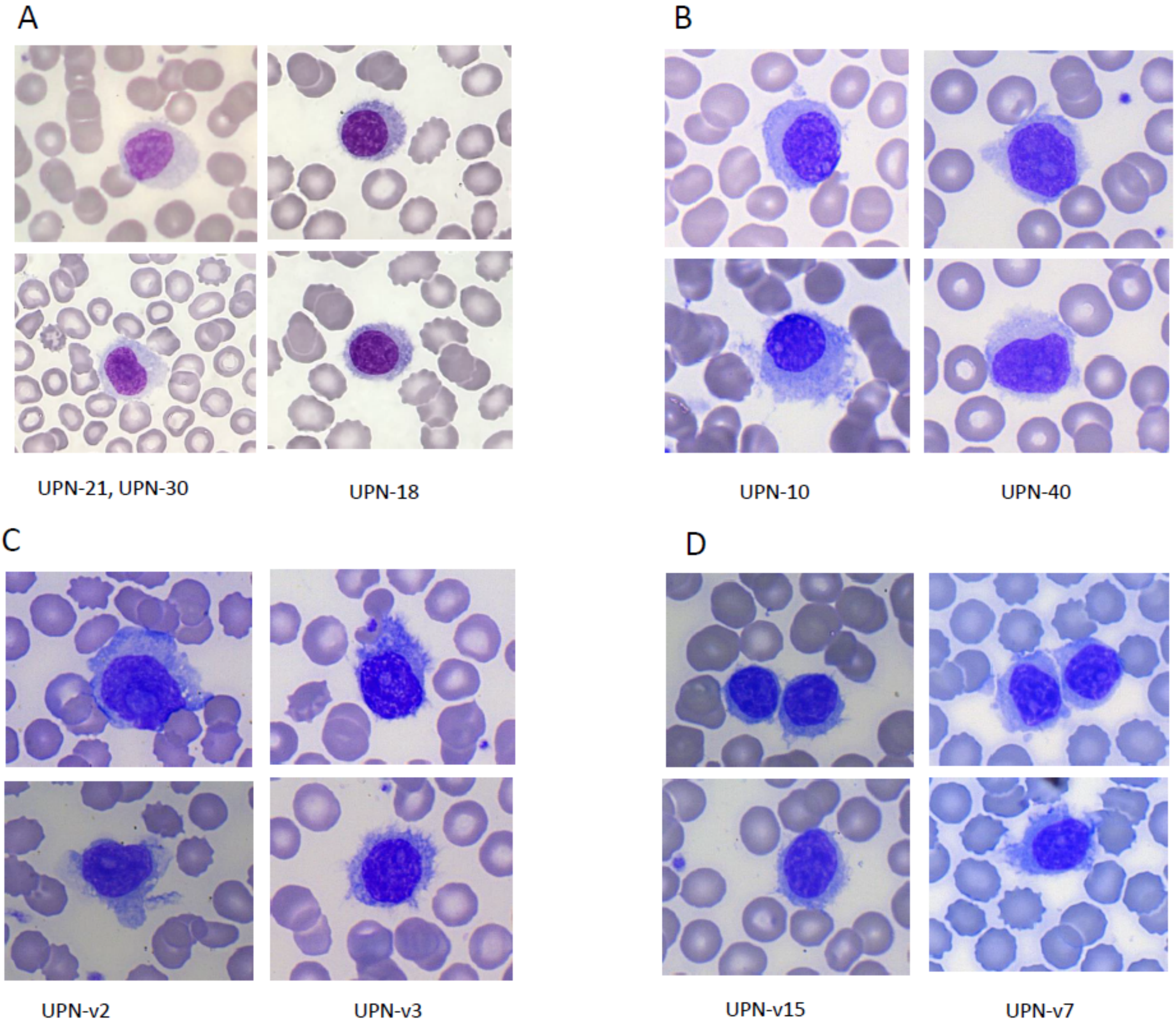

Figure S1. Representative cytomorphology of the different groups of HCL (cHCL *BRAF*^{V600E}, cHCL *BRAF*^{WT}, vHCL/SDRPL, HCL-like NOS). Blood smear, May–Grunwald–Giemsa staining, magnification × 1000.

A

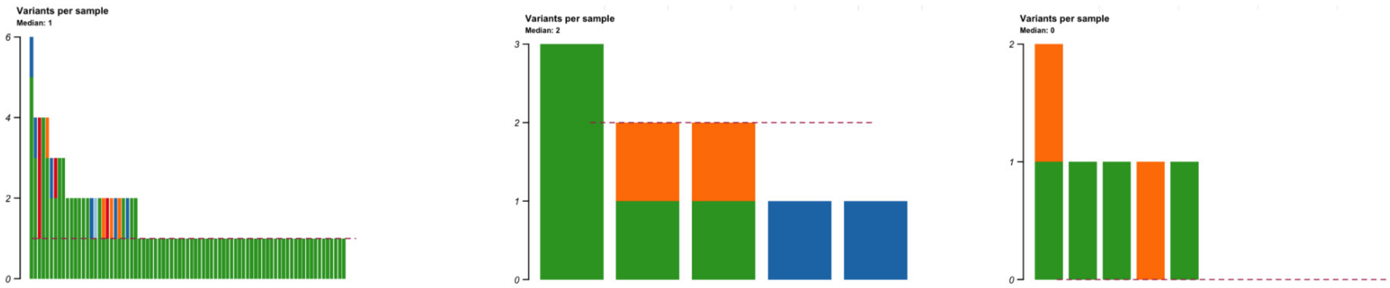

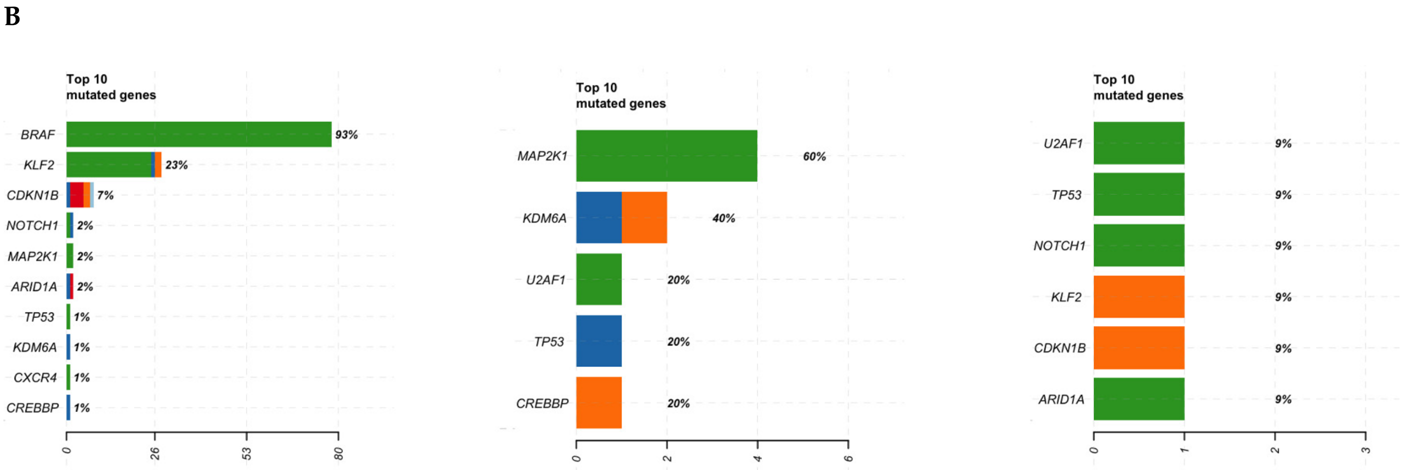

Figure S2. A: Representation of number of variants per sample of cHCL (*n* = 82), vHCL/SDRPL (*n* = 5) and HCL-like NOS (*n* = 11) individual patients. **B**: Top 10 mutated genes of cHCL (*n* = 82), vHCL/SDRPL (*n* = 5) and HCL-like NOS (*n* = 11) individual patients.

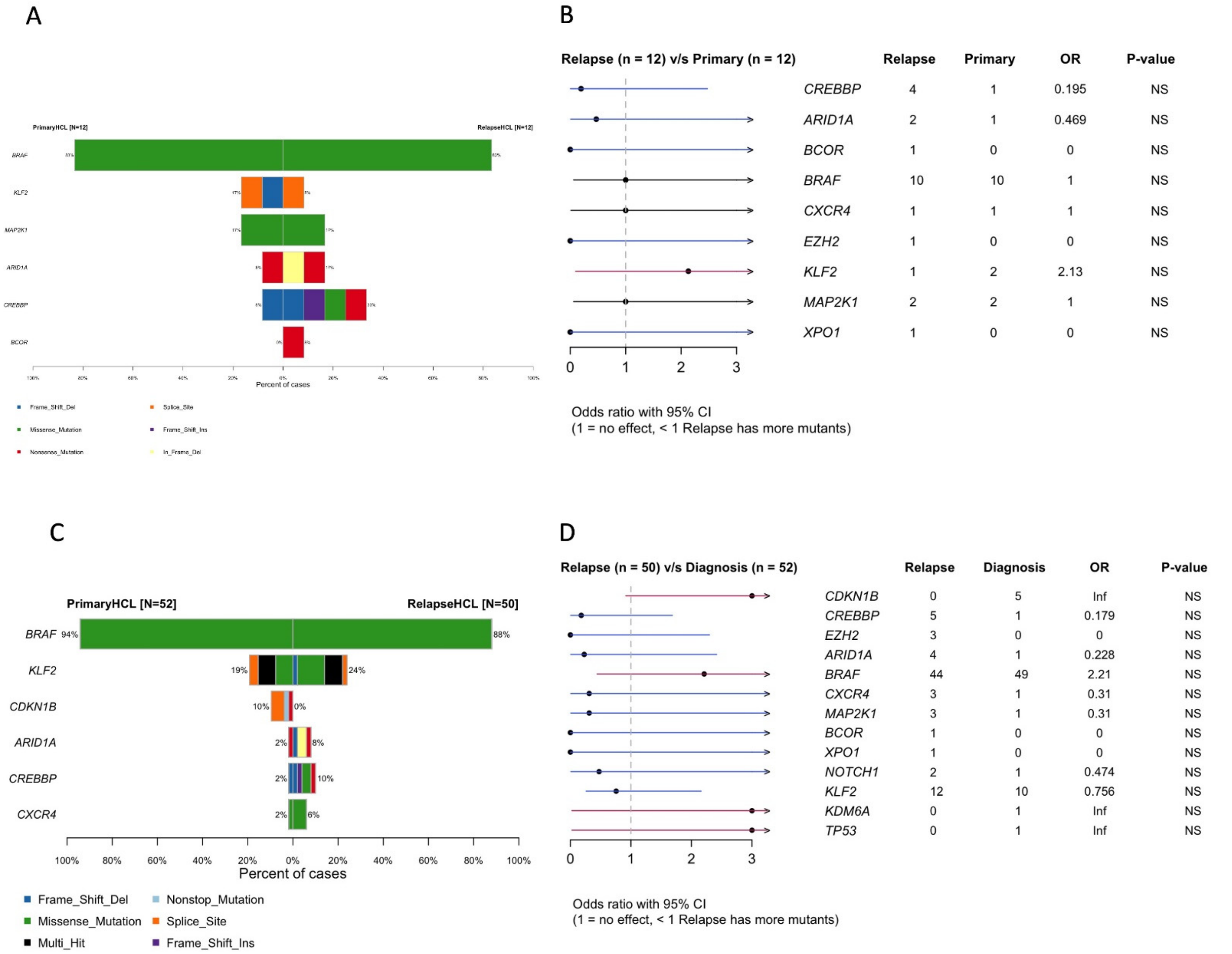

Figure S3. Representation of the mutational landscape of the sequential samples (*n* = 12 patients). **A**: Barplot. **B**: Forrest plot, representation of the mutational landscape of the whole cohort (*n* = 102 samples). **C**: Barplot. D: Forrest plot.

A

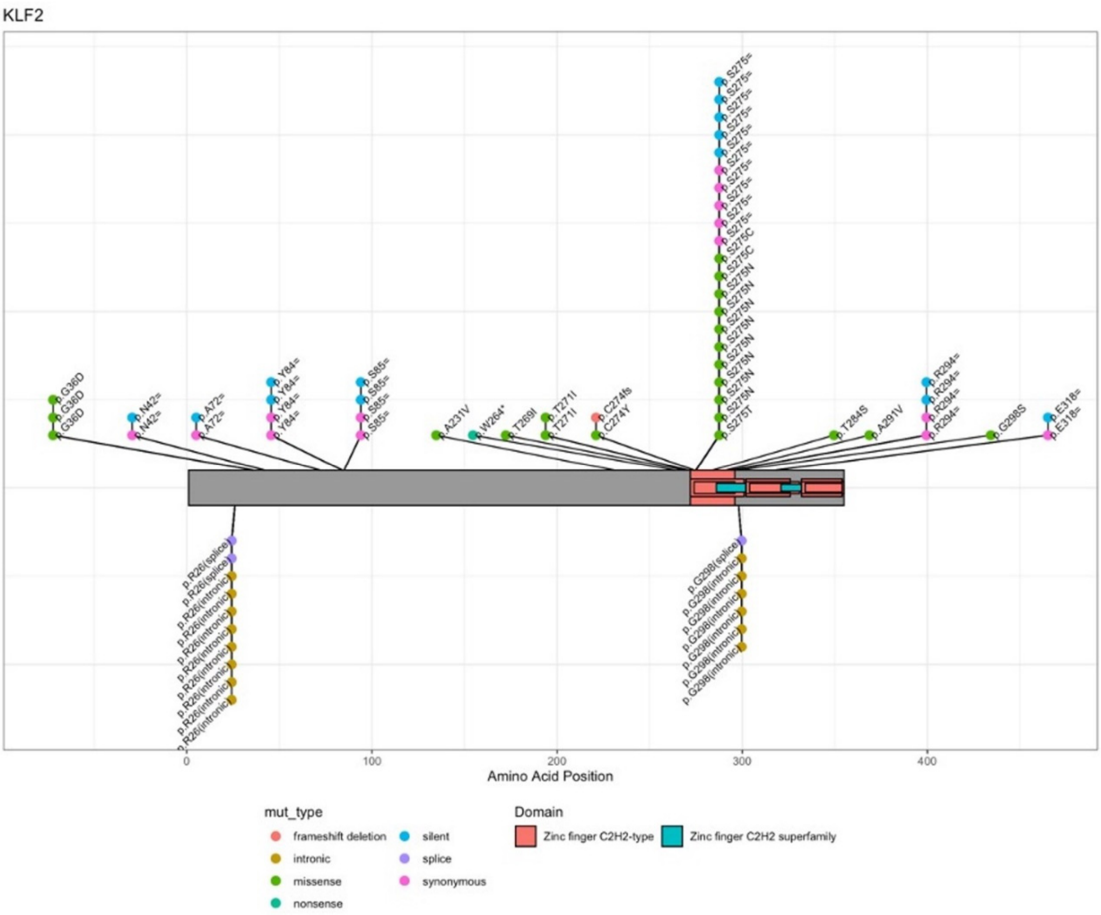

B

```
                             C
                             AT
                             AT
                             AT
                             AT
                             AT
                             AT
                 T           AT                         G
           T     T   T    A TAT                         T
799 AAACGCACCGCCACTCACACCTGCAGCTACGCGGGCTGCGGCAAGACCTACACCAAGAGTTCGC  862
267 -K--R--T--A--T--H--T--C--S--Y--A--G--C--G--K--T--Y--T--K--S--S--  287

             T        AT         A    A
863 ATCTGAAGGCGCATCTGCGCACGCACACAG // GTGAGAAGCCCTACCACTGCAACTGGGACG  922
288 H--L--K--A--H--L--R--T--H--T-- // G--E--K--P--Y--H--C--N--W--D--  307

                 A                 A        T
923 GCTGCGGCTGGAAGTTTGCGCGCTCAGACGAGCTCACGCGCCACTACCGAAAGCACACGGGCCA  986
308 G--C--G--W--K--F--A--R--S--D--E--L--T--R--H--Y--R--K--H--T--G--H  329

987 CCGGCCATTCCAGTGCCATCTGTGCGATCGTGCCTTCTCGCGCTCCGATCACCTGGCGCTGCAC 1050
330 --R--P--F--Q--C--H--L--C--D--R--A--F--S--R--S--D--H--L--A--L--H-  350

1051 ATGAAACGGCACATGTAG.............................................. 1068
351  -M--K--R--H--M--*-..............................................  355
```

Figure S4. A: Lolliplot of *KLF2* mutations of all variants, including intronic and synonymous mutations. Polymorphisms were excluded thanks to VAF that corresponded to tumor infiltration or sub-clonal mutations. B: Representation of mapping of mutations to the consensus sequence of the zinc finger domain of *KLF2* (267–350). Exon 2 is colored in blue and exon 3 in green. All point mutations identified in patients are shown above the reference sequence. Hot spot AID motifs (RCY) and C>T, G>A transition are highlighted in bold letters and violet.

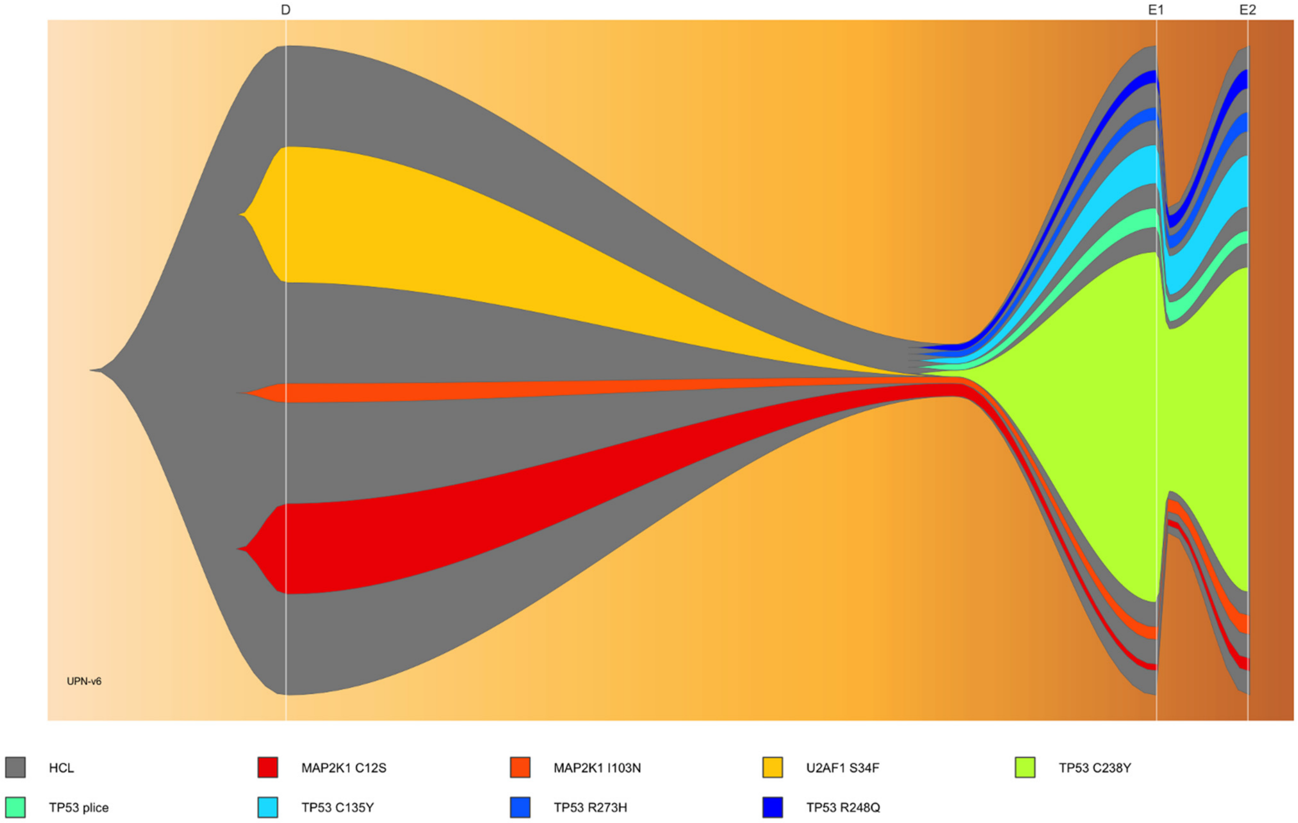

Figure S5. Fish plot representation of the clonal evolution of the mutations for UPN-v6. The patient did not receive any drug treatment during evolution of the disease. A splenectomy was carried out at E1. D: Diagnosis, E1: +143 months, E2: + 158 months.

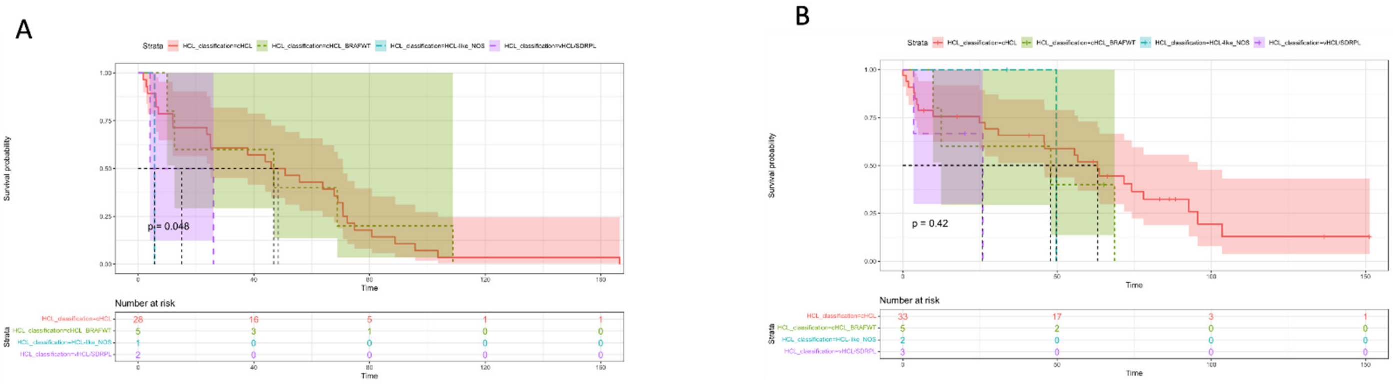

Figure S6. Survival analyses according to disease type. **A**: PFS and **B**: TTNT.
